# GC/MS Profiling and *Ex Vivo* Antibacterial Activity of *Salvadora persica* (Siwak) against *Enterococcus faecalis* as Intracanal Medicament

**DOI:** 10.1155/2021/6333867

**Published:** 2021-12-27

**Authors:** Nahla Ayoub, Nadia Badr, Saeed S Al-Ghamdi, Arwa Alzahrani, Rahaf Alsulaimani, Afnan Nassar, Rawabi Qadi, Ibtesam K. Afifi, Noha Swilam

**Affiliations:** ^1^Department of Pharmacology and Toxicology, Faculty of Medicine, Umm Al-Qura University (UQU), Makkah, Saudi Arabia; ^2^Department of Dental Biomaterials, Faculty of Dentistry, Umm Al-Qura University (UQU), Makkah, Saudi Arabia; ^3^Bachelor of Dental Surgery, Faculty of Dentistry, Umm Al-Qura University (UQU), Makkah, Saudi Arabia; ^4^Department of Preventive Dentistry, Public Health, Faculty of Dentistry, Umm Al-Qura University (UQU), Makkah, Saudi Arabia; ^5^Department of Basic and Clinical Oral Sciences, Faculty of Dentistry, Umm Al-Qura University (UQU), Makkah, Saudi Arabia; ^6^Department of Medical Microbiology and Immunology, Faculty of Medicine, Tanta University, Tanta, Egypt; ^7^Department of Pharmacognosy, Faculty of Pharmacy, The British University in Egypt (BUE), Cario, Egypt

## Abstract

**Introduction:**

*Salvadora persica* L. (*S. persica*, Siwak) has been used for many centuries as oral hygiene tools, particularly in Saudi Arabia. This study aimed to assess the effectiveness of *S. persica* petroleum ether extract (SPE) as an intracanal bactericidal for endodontic treatment against *Enterococcus faecalis*. Calcium hydroxide Ca(OH)_2_ gold standard intracanal medicament was used for comparison.

**Methods:**

The gas chromatography mass spectrometry (GC/MS) analysis was carried out to identify the components of SPE. First, the consistency of SPE was accomplished according to ANSI/ADA specification no 57. Forty-five single-rooted mandibular premolars were infected with that of *E. faecalis* suspension. Colony-forming units (CFU) were counted before the medicaments' application (CFU-1) and after seven days of their applications (CFU-2). Group I: SPE, Group II: positive control Ca(OH)_2_, and Group III: saline solution negative control. The microdilution method was applied to determine minimal inhibitory concentration (MIC) and minimal bactericidal concentration (MBC) of SPE.

**Results:**

Thirty-two compounds were identified (89.09%), with main components of benzyl isothiocyanate (BITC) (33.32%) and steroids (34%). CFU before and after using SPE and Ca(OH)_2_ recorded a statistically significant reduction in bacterial count (*P*=0.006) and (*P*=0.01), respectively. There was an insignificant difference between CFU after using SPE and Ca(OH)_2_ (*P*=0.210). On the contrary, comparing both medicaments with the negative control saline group resulted in significant differences, (*P*=0.001) and (*P*=0.007), respectively. Moreover, the equality of minimum bactericidal concentration (MBC) and minimum inhibitory concentration (MIC) of SPE is recorded.

**Conclusion:**

This finding could be referred to the high content of bactericidal BITC in synergism with other antimicrobial components, representing 70.71% of SPE. Thus, SPE is a good candidate as an intracanal medicament, which warrants further investigation.

## 1. Introduction

The ultimate goal of an infected root canal preparation, including cleaning, shaping, and using the disinfectant solution, is to entirely remove bacteria, their by-product, and pulpal remnants. The microorganisms in the root canal of the teeth are principally responsible for pulpal/periapical diseases. The primary intraradicular infections have been caused by many members of endodontic bacterial communities [1]. Some of them are persistent pathological bacteria, including *Streptococcus sanguinis, Streptococcus mutans, Enterococcus faecalis, Fusobacterium nucleatum, Porphyromonas gingivalis, and Prevotella intermedia* [2]. However, *E. faecalis* is significantly more associated with asymptomatic cases of primary endodontic infections. Furthermore, *E. faecalis* is much more likely to be found in cases of failed endodontic therapy [3]. The antibacterial intracanal medication is used to eradicate bacteria in adults and children's root canal systems. Accordingly, the pain and inflammation of pulpal and periapical tissues are reduced [4, 5]. This represents an optimal root canal disinfection protocol that guarantees a high success rate of root canal treatment [6–8]. The most commonly used intracanal medicament is Ca(OH)_2_ because of its significant bactericidal effect and inhibition of inflammatory exudates [8].

The foremost commonly utilized irrigants in endodontics are sodium hypochlorite (NaOCl), ethylene-diamine-tetra-acetic acid (EDTA), and chlorhexidine (CHX), which can cause destructive side effects. Intracanal medicament such as Ca(OH)_2_ causes collagen breakdown and leads to weakening of radicular dentin. Numerous plants are utilized as phytomedicines in dentistry since they have biological and antibacterial impacts. In endodontics, plants and their extracts can be utilized as irrigant and intracanal medicament to avoid the potential side effects caused by routine chemical agents [9].

*Salvadora persica* L. (*S. persica*. Siwak) is a plant whose roots, twigs, or stems have been used for many centuries as oral hygiene tools, particularly in Saudi Arabia. Using Siwak (tooth stick) for cleaning of mouth is advocated Islamic well-established beliefs [10].

Many studies have been demonstrated that extracts of *S. persica* possess various antiplaque, antiperiopathic, anticaries, anti-inflammatory, and antimycotic effects [11]. Extracts of *S. persica* as intracanal irrigant also proved significant antimicrobial activity on the oral pathogens in both *in vitro* and *in vivo* [12–14]. The present *in vitro* study was designed to assess the effect of an experimental intracanal medicament based on SPE against the most causative bacteria for endodontic pulpitis/periodontitis; *E. faecalis*. The gold standard intracanal medicament Ca(OH)_2_ was employed as positive control. The null hypothesis: there would be no difference between Ca(OH)_2_, the gold standard endodontic medicament, and SPE as an experimental intracanal medicament in bactericidal activity against *E. faecalis* for 7 days.

## 2. Materials and Methods

### 2.1. Materials

The materials used in this study were *S. persica* sticks brought from Riyadh region in Saudi Arabia, zinc oxide powder (zinc oxide; Prevest DenPro, Bari Brahmana, India), and Ca(OH)_2_ endodontic medicament (MetaPaste; META BIOMED, Chungcheongbuk-do, Republic of Korea).

The plant was identified by Dr. Talal Dahan, assistant professor of plant classification at Bisha University, Saudi Arabia, and a voucher specimen (# 19750) was deposited in properly labeled polythene bags for future reference at the Herbarium Centre, College of Pharmacy, Umm Al-Qura University (UQU), Saudi Arabia. The authors followed IUCN Policy Statement on Research Involving Species at Risk of Extinction.

### 2.2. Preparation of SPE

The fresh plant sticks of *S. persica* were freeze-dried and then ground to a fine powder using a commercially available food blender. The ground sample (500 g) was used to prepare the extract. Consequently, petroleum ether extract was prepared by cold percolating 500 g of dried powder of the plant sticks in one liter of petroleum ether for 72 h, and every 24 h fresh solvent was used. The solvent was removed and recovered in a rotary evaporator (BÜCHI Rotavapor RII; Büchi Labortechnik, Flawil, Switzerland) at 40°C using a BÜCHI vacuum pump. At the last stage, the oily extract was freeze-dried to ensure that solvents were removed to yield 20 ml of an oily material (SPE) with a powerful aromatic odor. SPE was kept in a brown screw-capped tube in a −20°C freezer until further analysis.

### 2.3. Gas Chromatography-Mass Spectrometry GC/MS Analysis

Mass spectra were recorded using Shimadzu GCMS-QP2010 (Kyoto, Japan) equipped with Rtx-5MS fused bonded column (30 m × 0.25 mm internal diameter i.d ×0.25 *µ*m film thickness) (Restek, USA) and a split-splitless injector. The initial column temperature was kept at 50°C for 3 min (isothermal), programmed to 300°C at a rate of 5°C/min, and kept constant at 300°C for 10 min (isothermal). Injector temperature was 280°C. Helium carrier gas flow rate was 1.37 ml/min. All the mass spectra were recorded applying the following conditions: (equipment current) filament emission current, 60 mA; ionization voltage, 70 eV; and ion source, 220°C. Diluted samples (1% v/v) were injected with split mode (split ratio) 1 : 15.

#### 2.3.1. Compounds Identification

The identification of compounds was performed based on their retention indices relative to a homologous series of *n*-alkanes (C8–C28) injected under the same conditions and matching their mass spectra with National Institute of Standards and Technology (NIST) (Gaithersburg, United States) and Wiley Library database (John Wiley & Sons, Hoboken, New Jersey, United States) as well as literature [15–19].

### 2.4. Collection of Extracted Teeth

A total sample size of 15 samples for each group was sufficient to reject the null hypothesis that the bactericidal activity for Ca(OH)_2_ and SPE was equal with probability (power) 0.8 as the true probability of success among the intervention group is 0.65. The Type I error probability associated with this test of this null hypothesis was 0.05. The sample size was calculated by G power program 3.1.9.2 (University of Düsseldorf, Düsseldorf, Germany). Forty-five single root premolars extracted teeth for periodontal or orthodontic reasons were obtained from the department of oral surgery at a different governmental and private hospital in Makkah city, Saudi Arabia. Teeth with previous endodontic therapy, fracture, crack, resorption, and root caries were excluded from the study. The present study followed the ethical guidelines for clinical investigation: ethical policy of the American Dental Association (ADA) regarding the use of human subjects in clinical research and approved by the Institutional Review Board (IRB), 21 January 2019, Faculty of Dentistry, Umm Al-Qura University, Saudi Arabia (IRB # 122-19).

### 2.5. Preparation of the Experimental Siwak-Based Extract Endodontic Medicament

#### 2.5.1. Determination of Consistency

The first intended procedure was to determine the appropriate powder/liquid mixing ratio (zinc oxide powder/SPE) for the experimental endodontic medicament. The purpose was to obtain a smooth and homogeneous material whose consistency is acceptable for clinical application. As well, the consistency should be analogous to the control endodontic medicament, commercial Ca(OH)_2_ paste (MetaPaste). The consistency was accomplished according to ANSI/ADA specification no# 57 [20]. Using a sterile stainless spatula and glass slab, the powder was added to the SPE in small increments until the cement was of ideal consistency: that is, until it formed a 2.5 cm string to connect the spatula to the glass plate when the spatula is lifted from the mix (one-inch string method). The paste was creamy in consistency but quite heavy. On obtaining the adequate consistency, the constituents' powder and liquid were quantified. It was found that when one scoop provided with zinc oxide powder mixed with one drop of SPE resulted in the required consistency [21].

#### 2.5.2. Preparation of Extracted Tooth

All forty-five single-rooted premolars were decoronated to standardize their root length. The working length was established to be 14-16 mm, and the roots was undergone rotary standardized instrumentation up to X3-ProTaper Next (DENTSPLY Tulsa Dental Specialties, Dentsply-Maillefer, Switzerland). An irrigant 2.5% sodium hypochlorite NaOCl (HYPOSOL; Prevest DenPro, Bari Brahmana, India) was used in each instrument and finally dried with size 25 absorbent paper points. Each root was placed in a closed test tube containing 3 ml of brain heart infusion (BHI) broth (BHI BROTH; SPML—Saudi Prepared Media Laboratory, Riyadh, Saudi Arabia) and sterilized by autoclaving at 121°C for 20 min and then incubated for 24 h at 37°C to confirm sterility by the absence of turbidity.

#### 2.5.3. Preparation of *E. faecalis* Suspension

*E faecalis* ATCC 29212 strain was grown on Bile Esculin agar media (BILE AESCULIN AGAR; SPML—Saudi Prepared Media Laboratory, Riyadh, Saudi Arabia) at 37°C for 24 h. After growth, bacterial suspension was prepared from grown bacterial colonies by inoculation in brain heart infusion (BHI) broth and adjusted to the optical density of approximately 1.5 × 10^8^ CFU/ml by comparing its turbidity to a 0.5 McFarland standard spectrophotometrically.

#### 2.5.4. Infection of Root Canals

Two ml of sterile BHI broth was removed from each root containing tube and replaced by 2 ml of the prepared bacterial suspension. The tubes were then closed and incubated at 37°C for 48 h.

#### 2.5.5. Colony-Forming Unit-1 (CFU-1)

After 48 h, predetermined contamination period of root canals was elapsed, each root was removed from the tube under complete aseptic precautions, and irrigated with sterile saline (100 *µ*l). Later, a dry sterile absorbent paper of point size 25 was inserted into the root canal and left for 5 min. Afterwards, these paper points were transferred individually to sterile test tubes, each containing 1 ml of sterile saline solution, vortexed for 30 seconds, and then four serial dilutions were made for each tube. Aliquots of 10 *µ*l of each dilution were plated onto Bile Esculin (BE) agar plates and incubated at 37°C for 24 h. The grown bacterial colonies were counted and multiplied by their dilution factor to represent CFU-1/ml.

#### 2.5.6. Ex Vivo Testing of Antimicrobial Activity of the Experimental Medicaments

The 45 decoronated teeth were randomly allocated into 3 equal groups (*n* = 15) by simple random sampling using random digit table depending on medicament used:

  Group I: experimental Siwak extract-based medicament
  Group II: Ca(OH)_2_ paste (positive control)
  Group III: sterile physiological saline as negative control group

Medicaments were placed into root canals with lentulo spiral (Dentsply-Maillefer, Swaziland) inserted to the full working length. The root canal foramina were sealed by sterile cotton pellets and the orifices closed by temporary restoration; then, the samples were kept in an incubator at 37°C for seven days.

#### 2.5.7. Colony-Forming Unit-2 (CFU-2)

Seven days later, each root was irrigated with sterile normal saline, and then the previous technique was repeated to obtain CFU-2.

#### 2.5.8. The Minimal Inhibitory Concentration (MIC) and Minimal Bactericidal Concentration (MBC) of SPE

The MIC of the SPE was determined in 96 multiwell microtiter plates using microdilution method [22] with minor modifications. SPE was adjusted to a concentration of 50 mg/ml in cation-adjusted Mueller Hinton broth medium, then pipetted 50 *µ*l of cation-adjusted Mueller Hinton broth medium into the first well of the plate, and 50 *µ*l of broth medium was distributed from the 1st to the 12th well of each row. Twofold serial dilution was achieved by transferring 50 *µ*l of scalar dilution from the first to the subsequent wells of each row. The final concentration of SPE adopted to evaluate antibacterial activity was included from 25 mg/ml to 0.003 mg/ml. Finally, 10 *µ*L of *E. faecalis* suspension was added to each well. Two row lines in each plate were used as controls: one row line with Ca(OH)_2_ as a positive control (in a serial dilution of 25–0.003 mg/mL). Plates were incubated at 37°C for 18–24 h. The lowest concentration at which no turbidity occurred was taken as the MIC value. Plates were analyzed individually to determine MIC, and the average MIC values from three repeats were taken in determination of the final MIC values for each extract to ensure accuracy and reproducibility. To determine the MBC of SPE, 50 *μ*l of the solution was removed from the well before the MIC well and the well after the MIC well. The solution was inoculated into tryptic soy agar plate and incubated at 37°C for 24 h.

### 2.6. Statistical Analysis

Data were collected, tabulated, and statistically analyzed using Statistical Package for Social Science (SPSS v.20, IBM. Released 2020. IBM SPSS Statistics for Windows, Version 27.0. Armonk, NY: IBM). *P* < 0.05 was considered as a level of significance. The mean and standard deviation were tabulated and statistically analyzed using one-way ANOVA test regarding the CFU-1 of the three tested groups before using intracanal medication. Regarding the CFU-2 of three groups, the mean and standard deviation were analyzed using one-way nonparametric ANOVA (Kruskal–Wallis). Finally, the antimicrobial effect of different tested medications on *E. faecalis* was tabulated and statistically analyzed using paired *t*-test.

## 3. Results

### 3.1. Chemical Composition of SPE

Analysis of SPE by GC and GC/MS revealed that SPE contained 32 compounds, of which benzyl isothiocyanate (33.32%), *γ*-sitosterol (25.76%), stigmasterol (5.92%), *β*-sitosterol acetate (2.28%), *n*-hexadecanoic acid (4.27%), and (*Z*)-11-octadecenoic acid (3.16%) were found to be the major constituents. Compounds are listed in order of their elution times on Rtx-5MS column in Table 1.

### 3.2. SPE Endodontic Therapy

The statistical analysis of the CFU-1 of three tested groups before using intracanal medications (SPE and Ca(OH)_2_) was tabulated in Table 2. It was evident that there were no significant differences in bacterial counts among them (*P*=0.359). Table 2 shows the statistical analysis using paired *t*-test of the number of the CFU of *E. faecalis* before and after applying the investigated medicaments at the root canal lumens; CFU-1 and CFU-2, respectively. The results showed a significant difference between CFU-1 and CFU-2 either in using SPE (*P*=0.006) or Ca(OH)_2_ (*P*=0.011). On the other hand, the bacterial count of the negative control group showed a nonsignificant difference between CFU-1 and CFU-2 (*P*=0.438). Finally, the bacterial count's significant difference among the three tested groups was compared using one-way nonparametric ANOVA. The values were insignificantly different between bacterial counts after using SPE and Ca(OH)_2_ (*P*=0.210). On the contrary, comparing both medicaments with the negative control saline group resulted in significant differences (*P*=0.001) and (*P*=0.007), respectively.

### 3.3. MIC and MBC of SPE

The MIC of SPE and Ca(OH)_2_ were measured to be 3.5 and 4.2 mg/ml, respectively. No growth in the MIC well and the well before that and bacterial growth in the well after the MIC well indicated equality of MBC and MIC of SPE.

## 4. Discussion

The chemomechanical cleaning and shaping of the root canals of the teeth are essential for the success of endodontic therapy [23, 24]. Yet, intracanal medication is a crucial procedure during root canal treatment where the probability of complete eradication of bacteria is questionable [25].

It is well documented that the use of biocompatible intracanal medications possessing antimicrobial properties between appointments destroys and eradicates bacteria in the root canal system [26]. It was proven that *E. faecalis* is the most predominant bacteria found in the infected root canal [27]. Unfortunately, this species has the ability to survive in hard environment with deprived nutrients and alkaline pH reaching up to 11.5 that implies a challenge to be completely exterminated from root canal system [28, 29].

Calcium hydroxide Ca(OH)_2_ is the gold standard intracanal medication used in the treatment of infected root canals, referring to its well-known antibacterial features and ease of application [30, 31]. The effect of both SPE and Ca(OH)_2_ against *E. faecalis* was considered in the presence of a negative control group saline, to specify the antimicrobial effect. The antimicrobial effect of the experimental medicament SPE insignificantly surpasses that of Ca(OH)_2_ (*P*=0.210), as shown in Table 2. In the present investigation, SPE experimental intracanal medicament (Group l) proved an effective eradication of *E. faecalis*. This finding was in agreement with the results reported about the usefulness of SPE as endodontic irrigant against *E. faecalis* [32]. As well, it was evidenced that *S. persica* has an inhibitory effect on the growth of the oral pathogen [33]. The MIC value of SPE was in agreement with those reported for aqueous and methanol extracts of *S. persica* against *E. faecalis* [34] and for Ca(OH)_2_ [35]. MIC of SPE is recorded in the current study for the first time.

In this study, the chemical profile representing 32 compounds of SPE was identified by GC/MS (89.09%) and listed in Table 1. The major component was found to be benzyl isothiocyanate. This compound was reported as the most robust antibacterial component with high bactericidal activity against Gram-negative periodontal pathogens [36]. The potent antibacterial effect of the experimental medicament, as shown in Table 2, suggested that SPE targeted the bacterial membrane, whereas BITC has both lipophilic and electrophilic properties. It is reported that BITC penetrates through the outer bacterial membrane and possibly interfered with the bacterial redox systems, thus hampering the bacterium's ability to maintain its membrane potential. Such an effect of BITC has been demonstrated for mitochondrial membranes [37].

In addition, there are *γ*-sitosterol (25.76%), *n*-hexadecanoic acid (4.27%), and (*Z*)-11-octadecenoic acid (3.16%) that are reported as potent broad-spectrum antimicrobials [38–42]. Moreover, SPE has antimicrobial effect against both Gram-positive and Gram-negative bacteria due to the presence of carvacrol (0.57%) confirmed by Memar *et al*. (2017) [43]. SPE components in Table 1 show the presence of *n*-tetradecane (0.21%), *n*-hexadecane (0.13%), 11-octadecenoic acid (3.16%), squalene (0.17%), stigmasterol (0.12%), sitosterol (2.28%), and sitostenone (0.54%), and these components are potent antibacterial agents [44–48]. Therefore, SPE's strong bactericidal effect is attributed to the antimicrobial components that represents a percentage of 70.71% of the extract. It is worthy to mention that some components with anti-inflammatory and antioxidant effects are identified as oleic acid (1.95%), 11-octadecenoic acid (3.16%), 2,2′-methylenebis (6-*tert*-butyl-4-methylphenol) (0.13%), squalene (0.17%), *n*-benzyloleamide (0.53%), *n*-benzylpalmitamide (0.36%) stigmasta-5, 22-dien-3-ol (0.65%) lupeol (1.24%), and hexadecanoic acid, ethyl ester (0.13%) [46, 47, 49].

Based on the obtained results, the null hypothesis is accepted as the experimental intracanal medicament SPE, a novel trial in the field of endodontic treatment, has nonsignificant difference in antimicrobial activity comparing to the gold standard; Ca(OH)_2_ against *E. faecalis*. However, further studies are suggested to explicate SPE effect against other reported endodontic pathogens. Moreover, the limitations of this study as the assessment of the activity against the biofilm formation that resembles the clinical situation should be investigated after the evaluation of the effect of SPE in an in *vivo* study. SPE comprises potent antimicrobial and anti-inflammatory components as identified by GC/MS. Thus, it could be claimed that it will be a robust intracanal medicament. However, its anti-inflammatory effect is still under investigation by the authors.

## 5. Conclusion

Within the limitations of this *Ex vivo* study, SPE can efficiently eradicate *E. faecalis* from the root canal system by many antimicrobial components. It could be used as a good alternative to calcium hydroxide in endodontic therapy; however, further studies are recommended to investigate its efficacy in an *in vivo* assessment and inhibitory effects on biofilm formation before engaging it in regular dental practice.

## Figures and Tables

**Table 1:** The chemical profile of SPE.

Peak #	*R*_*t*_	Component	Molecular formula	*RI*_*exp*_^*a*^	*RI*_*lit*_^*b*^	Content (%)	Identification^c^
1.	18.35	Carvacrol	C_10_H_14_O	1288	1288	0.57	MS, KI
2.	20.47	Benzyl isothiocyanate	C_8_H_7_NS	1361	1359	**33.32**	MS, KI
3.	21.14	*n*-Tetradecane	C_14_H_30_	1384	1400	0.21	MS, KI
4.	23.76	*n*-Pentadecane	C_15_H_32_	1485	1500	0.38	MS, KI
5.	26.24	*n*-Hexadecane	C_16_H_34_	1582	1600	0.13	MS, KI
6.	34.40	*n*-Hexadecanoic acid (palmitic acid)	C_16_H_32_O_2_	1948	1946	**4.27**	MS, KI
7.	34.91	Hexadecanoic acid ethyl ester	C_18_H_36_O_2_	1972	1975	0.13	MS, KI
8.	36.12	Cyclic octaatomic sulphur	S_8_	2034	2055	0.99	MS, KI
9.	37.86	Oleic acid	C_18_H_34_O_2_	2130	2130	**1.95**	MS, KI
10.	37.92	(*Z*)-11-Octadecenoic acid	C_18_H_34_O_2_	2133	2117	**3.16**	MS, KI
11.	38.15	(*Z,Z*)-9,12-Octadecadienoyl chloride (linoleic acid chloride)	C_18_H_31_ClO	2146	2139	0.37	MS, KI
12.	38.24	(*Z*)-9-Octadecenoic acid ethyl ester (oleic acid ethyl ester)	C_20_H_38_O_2_	2151	2168	0.45	MS, KI
13.	38.36	9-Octadecenoic acid ethyl ester	C_20_H_38_O_2_	2157	2171	0.14	MS, KI
14.	42.84	*p-*Cresol, 2,2′-methylene bis [6-tert-butyl-	C_23_H_32_O_2_	2403	2398	0.13	MS, KI
15.	48.95	Squalene	C_30_H_50_	2796	2814	0.17	MS, KI
16.	49.51	N-Benzylpalmitamide	C_23_H_39_NO	2832	2880	0.36	MS
17.	51.09	Stigmasta-3,5-diene	C_29_H_48_	2934	—	0.77	MS
18.	51.69	Stigmasterol acetate	C_31_H_50_O_2_	2972	2879	0.12	MS, KI
19.	51.87	3*β*-Acetoxystigmasta-4,6,22-triene	C_31_H_48_O_2_	2984	-	0.24	MS
20.	52.06	*n*-Benzyloctadecenamide (*n*-benzyloleamide)	C_25_H_41_NO	2996	2991	0.53	MS, KI
21.	52.17	Stigmasta-5,22-dien-3-ol, acetate	C_31_H_50_O_2_	3003	-	0.65	MS
22.	52.47	Clionasterol acetate	C_31_H_52_O_2_	3022	-	0.50	MS
23.	52.66	Stigmastan-3,5,22-triene	C_29_H_46_	3034	2981	1.89	MS, KI
24.	52.94	*β*-Sitosterol acetate	C_31_H_52_O_2_	3052	-	**2.28**	MS
25.	53.28	Cholesterol	C_27_H_46_O	3074	3075	0.66	MS, KI
26.	54.89	Campesterol	C_28_H_48_O	3177	3131	0.70	MS
27.	55.40	Stigmasterol	C_29_H_48_O	3210	3213	**5.92**	MS, KI
28.	56.44	*γ*-Sitosterol	C_29_H_50_O	3277	3290	**25.76**	MS, KI
29.	57.47	Unidentified	-	3344	-	**3.57**	-
30.	58.21	3,5-Stigmastadien-7-one	C_29_H_46_O	3391	-	0.56	MS
31.	59.04	Sitostenone	C_29_H_48_O	3444	3435	0.54	MS, KI
32.	60.19	Lupeol	C_30_H_50_O	3518	3451	1.24	MS
	% total identified			89.09			

Compounds are listed in order of their elution times on Rtx-5MS column. ^a^*RI*_*exp*_, retention index determined experimentally relative to C8 – C28 *n*-alkanes on Rtx-5MS column; ^b^*RI*_*lit*_, published retention indices; ^c^Identification, was based on comparison of the compounds' mass spectral data (MS) and retention indices (RI) with those of NIST Mass Spectral Library (2017), Wiley Registry of Mass Spectral Data 8^th^ edition and literature. Bold shows major components.

**Table 2:** The descriptive statistical analysis of the *E. faecalis* count before (CFU-1) and after (CFU-2) the application of the medicaments.

Bacterial count groups	CFU-1 Mean ± SD	CFU-2 Mean ± SD
Group I Siwak	48.9 ± 53.7 *∗* 10^4^	0.0250 ± 0.1 *∗* 10^4^
Group II Ca(OH)_2_ (+ve control)	51.6 ± 68.4 *∗* 10^4^	0.07 ± 0.25 *∗* 10^4^
Group III Saline (−ve control)	56.3 ± 59 *∗* 10^4^	50 ± 115 *∗* 10^4^

## Data Availability

The data that support the findings of this study are openly available from the corresponding author on reasonable request.

## Supplementary Materials

Supplementary Materials S1: *Salvadora persica plants*' sticks. S2: *Salvadora persica* pet-ether extract. S3: prepared 45 extracted teeth. S4: prepared 45 extracted teeth in BHI tubes and prepared for incubation at 37°C for 48 hours. Two ml of sterile BHI broth was removed from each root-containing tube and replaced by 2 ml of the prepared bacterial suspension. The tubes were then closed and incubated at 37°C for 48 h. S5: bile esculin (BE) agar showing a high number of growing colonies of *E. faecalis* before application of medicaments. S6: BE agar showing no growing colonies of *E. faecalis* after application of Siwak as a medicament (CFU2). S7: BE agar showing a few number of growing colonies of *E. faecalis* after application of Ca(OH)_2_ (CFU2). S8: BE agar showing a high number of growing colonies of *E. faecalis* after using saline (CFU2). S9: GC/MS total ion chromatogram (TIC) of *Salvadora sp.* petroleum ether extract. GCMS revealed the identification of 32 compounds from SPE by comparing their mass spectra with the National Institute of Standards and Technology (NIST) (Gaithersburg, United States) and Wiley library database (John Wiley & Sons, Hoboken, New Jersey, United States), as well as the literature.
