# Supplementary material for: GC/MS Profiling and Ex Vivo Antibacterial Activity of Salvadora persica (Siwak) against Enterococcus faecalis as Intracanal Medicament
Source: Evid Based Complement Alternat Med. 2021 Dec 27;2021:6333867. doi: 10.1155/2021/6333867 (PMC8723863; doi:10.1155/2021/6333867)
Supplement: Supplementary Materials — S1: Salvadora persica plants' sticks. S2: Salvadora persica pet-ether extract. S3: prepared 45 extracted teeth. S4: prepared 45 extracted teeth in BHI tubes and prepared for incubation at 37°C for 48 hours. Two ml of sterile BHI broth was removed from each root-containing tube and replaced by 2 ml of the prepared bacterial suspension. The tubes were then closed and incubated at 37°C for 48 h. S5: bile esculin (BE) agar showing a high number of growing colonies of E. faecalis before application of medicaments. S6: BE agar showing no growing colonies of E. faecalis after application of Siwak as a medicament (CFU2). S7: BE agar showing a few number of growing colonies of E. faecalis after application of Ca(OH)2 (CFU2). S8: BE agar showing a high number of growing colonies of E. faecalis after using saline (CFU2). S9: GC/MS total ion chromatogram (TIC) of Salvadora sp. petroleum ether extract. GCMS revealed the identification of 32 compounds from SPE by comparing their mass spectra with the National Institute of Standards and Technology (NIST) (Gaithersburg, United States) and Wiley library database (John Wiley & Sons, Hoboken, New Jersey, United States), as well as the literature. [file 6333867.f1.pdf]

The Effectiveness of *Salavadora persica* (Siwak) Petroleum Ether Extract as an Intracanal Medicament used in Endodontic Therapy: An *in vitro* Study

Nahla Ayoub[1*§], Nadia Badr[2], Saeed S Al-Ghamdi[1§], Arwa Alzahrani[3], Rahaf Alsulaimani[3], Afnan Nassar[4], Rawabi Qadi[1§], Ibtesam K. Afifi[5,6‡] Noha Swilam[7‡]

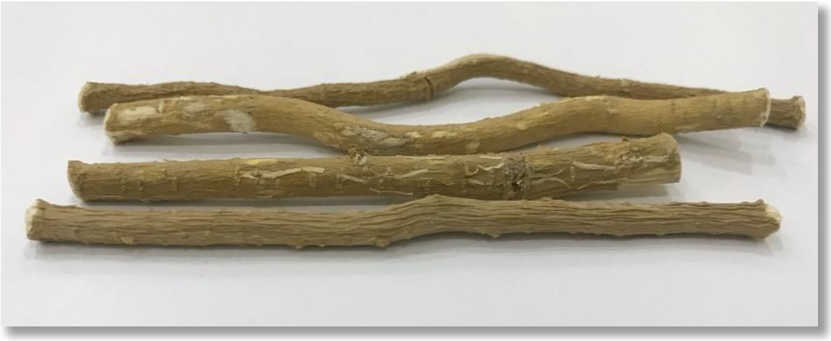

S1: ***Salvadora persica*** plants' sticks

The root sticks of cultivated *Salvadora persica* SP (namely Miswak, Siwak or Arak) plant were collected from Riyadh

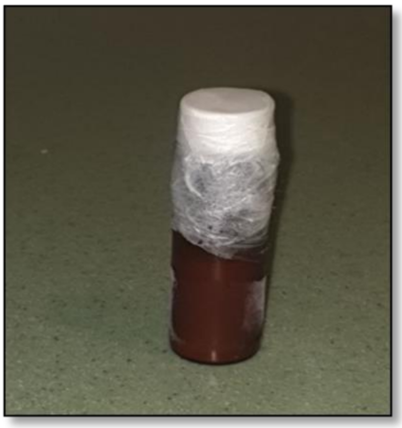

S2: ***Salvadora persica*** pet-ether Extract

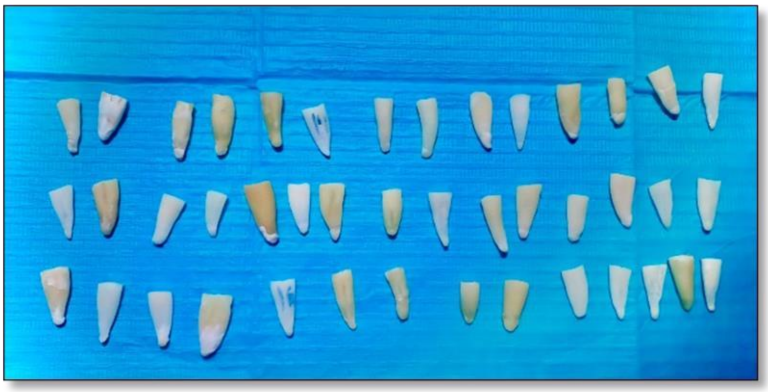

S3: Prepared 45 extracted

The fresh freeze-dried plant sticks of SP were extracted with petroleum ether extract by cold percolating 500 g of dried powder of the plant sticks in one liter of petroleum ether for 72 h, and every 24 h fresh solvent was used.

Two ml of sterile BHI broth was removed from each root containing tube and replaced by 2 ml of the prepared bacterial suspension. The tubes were then closed and incubated at 37 °C for 48 h.

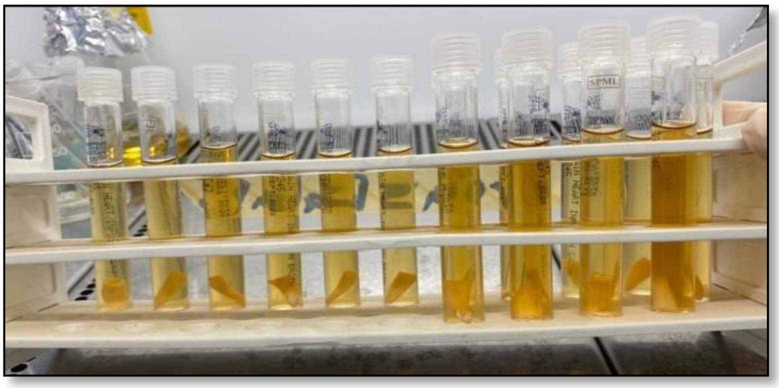

S4: Prepared 45 extracted teeth in BHI tubes and prepared for incubated at 37°C for 48 hours. Two ml of sterile BHI broth was removed from each root containing tube and replaced by 2 ml of the prepared bacterial suspension. The tubes were then closed and incubated at 37 °C for 48 h.

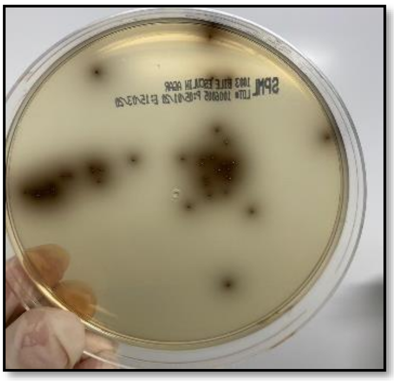	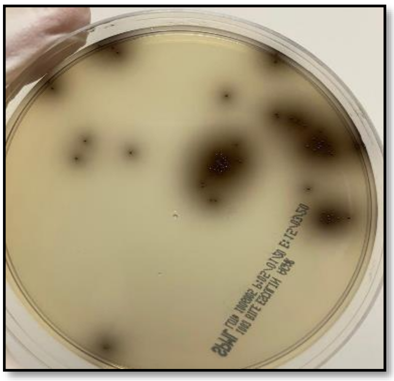	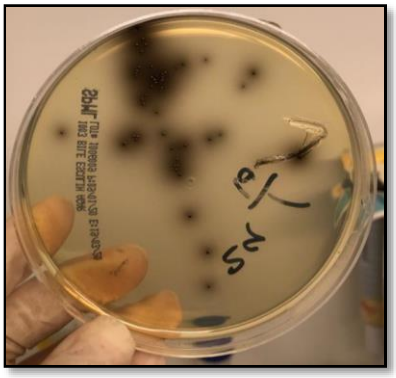
**S5**: Bile esculin (BE) agar showing high number of growing colonies of ***E. faecalis*** before application of medicaments		

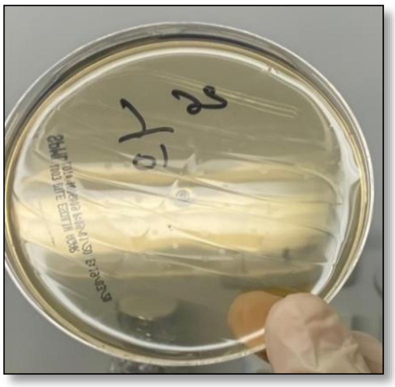	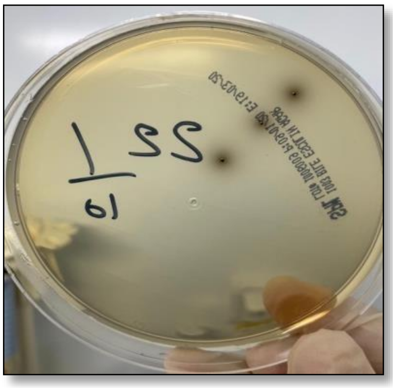	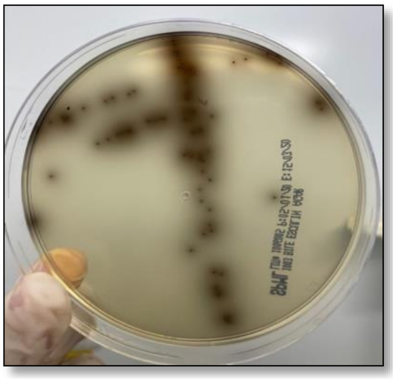
**S6:** BE agar showing no growing colonies of ***E. faecalis*** after application of Siwak as medicament (CFU2)	**S7:** BE agar showing few number of growing colonies of ***E. faecalis*** after application of $Ca(OH)_2$ (CFU2).	**S8:** BE agar showing high number of growing colonies of ***E. faecalis*** after using saline (CFU2)

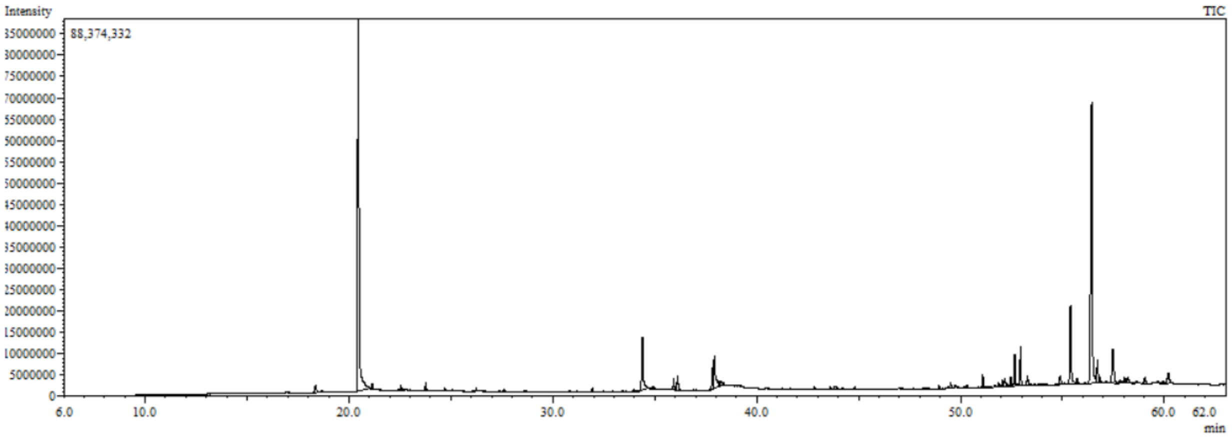

S9: GC/MS total ion chromatogram (TIC) of *Salvadora* sp. petroleum ether extract.

GCMS revealed the identification of 32 compounds from SPE by comparing their mass spectra with National Institute of Standards and Technology (NIST) (Gaithersburg, United States) and Wiley library database (John Wiley & Sons, Hoboken, New Jersey, United States) as well as literature
